# Assistive Technologies for Pulse-Based Tele-Practice in Traditional Chinese Medicine: Two-Phase, Dual-Method Feasibility and Pilot Study

**DOI:** 10.2196/77740

**Published:** 2026-03-06

**Authors:** Jia Yin Ruan, Shucheng Chen, Minru Wu, Shuhan Li, Yuan Shan Ho, Ka Lun Fan, Wing Leung Chow, Leung Chiu, Wing Fai Yeung

**Affiliations:** 1 School of Nursing, the Hong Kong Polytechnic University, GH506, Hung Hom, Kowloon, Hong Kong SAR, China (Hong Kong), 852 2766 4151; 2 New York University Rory Meyers College of Nursing, New York, NY, United States; 3 Department of Applied Data Science, Hong Kong Shue Yan University, Hong Kong SAR, China (Hong Kong); 4 Logistics and Supply Chain MultiTech R&D Centre, Hong Kong SAR, China (Hong Kong); 5 Research Centre for Chinese Medicine Innovation, Hong Kong SAR, China (Hong Kong)

**Keywords:** traditional Chinese medicine, tele-practice, pulse sensing, pulse regeneration, wireless network

## Abstract

**Background:**

Pulse palpation is essential for accurate traditional Chinese medicine diagnosis. However, this tactile-sensory-dependent technique is not feasible in teleconference, leaving traditional Chinese medicine underserved by conventional tele-practice frameworks. To address this, we developed an Assistive Pulse Data Collection (APDC; Logistics and Supply Chain MultiTech R&D Centre) device.

**Objective:**

This study aimed to evaluate feasibility and to preliminarily examine the Chinese medicine practitioners (CMPs) agreement on real-person pulses and machine-generated pulses and collect users’ feedback.

**Methods:**

Following World Health Organization guidelines for piloting new medical technologies, a 2-phase, dual-method evaluation was conducted. In the feasibility phase, 10 participants’ pulses were recorded using the APDC device. Overall, 5 CMPs evaluated the participants’ and machine-generated pulses using a 5-point Likert scale (1=“Strongly Disagree” to 5=“Strongly Agree”). In the pilot phase, 10 CMPs assessed pulses from 42 participants and refined the regenerated outputs for comparison. Quantitative and qualitative feedback from CMPs and participants was collected.

**Results:**

CMPs evaluated machine-regenerated pulses across 4 parameters: frequency (mean agreement score 4.1, SD 0.6), rhythm (mean 3.8, SD 0.6), width (mean 2.7, SD 0.9), and force (mean 2.4, SD 1), indicating moderate-to-high agreement for frequency or rhythm but lower consensus for width and force. Following device refinements (eg, adjustable armrest, pressure calibration, and pulse algorithms), the pilot phase CMPs’ agreement improved for frequency (mean 4.3, SD 0.7) and rhythm (mean 4, SD 0.8), while width (mean 3, SD 0.9) and force (mean 2.8, SD 0.9) remained suboptimal. CMPs reported enhanced clarity (mean 4.3, SD 1) but persistent inefficiency (mean 2.5, SD 0.5) and neutral satisfaction (mean 2.6, SD 0.5). Participants maintained favorable ratings for comfort (mean 4, SD 0.7), easy to use (mean 3.9, SD 0.8), and high satisfaction (mean 4, SD 0.7).

**Conclusions:**

The APDC device is feasible and enables CMPs to take remote pulse feature assessment, but further optimization of pulse force and width is warranted.

## Introduction

The COVID-19 pandemic has profoundly disrupted global health care systems, compelling a rapid shift toward tele-practice to mitigate barriers to in-person care [1,2]. While virtual consultations have proven effective for many medical disciplines, their application faces significant challenges in practices requiring tactile diagnostic methods, such as traditional Chinese medicine (TCM) [3]. A major barrier to tele-practice in TCM was the inability to conduct physical examinations, particularly pulse examinations on patients [4]. In TCM, pulse palpation is a diagnostic cornerstone where practitioners assess the depth, rhythm, and waveform of the radial artery to identify pathological patterns [5] and treatment formulation [6-8], which is irreplaceable for accurate TCM pattern diagnosis [9]. This tactile-sensory–dependent technique is not feasible in teleconference, leaving TCM underserved by conventional tele-practice frameworks. To bridge this gap, innovative solutions are urgently needed to replicate tactile diagnostic interactions remotely. Emerging research proposes robotic systems capable of capturing, quantifying, and recreating pulse waveforms through haptic interfaces, thereby enabling a remote pulse diagnosis [9]. Such technology could not only address pandemic-induced limitations but also expand access to TCM care in underserved regions, underscoring its transformative potential for both tele-practice and traditional practice.

Various sensor technologies have been used for the noninvasive monitoring of radial artery pulses. Currently, the main types of pulse sensors include pressure sensors, photoelectric sensors, and ultrasonic Doppler sensors. Each type has its distinct mechanisms and characteristics. Pressure sensors directly measure the radial artery pulse using piezoelectric, piezoresistive, or piezocapacitive sensors: piezoelectric sensors offer high repeatability and heat resistance but cannot detect static pressure [10-12]; piezoresistive sensors, known for high sensitivity and mature technology, are the most widely used [13,14]; piezocapacitive sensors provide accurate dynamic measurements but exhibit nonlinear characteristics [15,16]. In contrast, photoelectric sensors detect variations in light reflected from blood vessels, converting them into electrical signals [17,18]. While they are less affected by interference, they require a radiation source that complicates the circuitry. In contrast, ultrasonic Doppler sensors analyze reflected ultrasound waves to derive pulse signals from changes in blood flow speed and vessel volume, offering rich data but at a higher cost than the other types [19,20]. All 3 devices primarily focus on detecting and digitizing pulse data. However, in clinical practice, these digitized pulse readings are not easily interpretable by Chinese medicine practitioners (CMPs). Therefore, a device that can convert the collected pulse data back into a pulse format resembling human pulse features that is suitable for CMPs’ assessment is critically important for clinical practice.

To fill this critical gap in TCM tele-practice, we developed an Assistive Pulse Data Collection (APDC) device (Logistics and Supply Chain MultiTech R&D Centre, Hong Kong SAR). The APDC device integrates a high-fidelity pulse sensor to capture radial artery waveforms, digitizes key pulse features (eg, rhythm, frequency, and amplitude), and transmits data wirelessly to a robotic regeneration interface. Unlike conventional telemedicine platforms that are limited to audiovisual communication, this system enables TCM practitioners to remotely palpate and interpret pulse waveforms through haptic feedback, thereby replicating the tactile dimension essential for accurate pattern diagnosis. By bridging the sensory disconnect inherent in virtual consultations, the APDC device has the potential to enhance diagnostic precision and expand access to TCM care in remote areas and pandemic-restricted settings.

Validating such a device is imperative to ensure its accuracy, reliability, and safety for clinical use. It is essential to confirm that the device can accurately capture and reproduce the pulse features, as any discrepancies could lead to misdiagnosis and inappropriate treatment. We, therefore, conduct this feasibility and pilot study to evaluate the feasibility and to preliminarily examine the CMPs' agreement on real-person pulses and machine-generated pulses, and to collect CMPs’ and participants’ feedback on the device. This dual-method evaluation aligns with World Health Organization guidelines for piloting novel medical technologies, ensuring both technical accuracy and user experience are prioritized [21].

## Methods

### Study Design

The study consisted of 2 phases, namely the feasibility phase (phase 1) and the pilot phase (phase 2). The reporting of this study adhered to the guidelines of the CONSORT (Consolidated Standards of Reporting Trials) statement: extension to randomized pilot and feasibility trials [22].

### Phase 1: Feasibility

This phase aimed to preliminarily assess the feasibility of the APDC device in collecting participants’ pulse features and regenerating the pulse for CMPs. CMP’s rating of the agreement on the likeliness between participants’ and regenerated pulse was also preliminarily assessed. User experiences from both CMPs and participants were collected for the team to refine the setting of the APDC Device. The CMPs and participants were recruited from 20 to 28 November 2024.

### Procedure

The study procedure was conducted in the following steps:

1. The participant’s right-hand pulse features were recorded using the APDC Device.
2. Five CMPs assessed the participant’s pulse and then the pulse regenerated from the APDC Device.
3. The CMPs rated the agreement, using a predefined record form on the likeliness between the participants’ pulse and APDC-regenerated pulse, in the respects of frequency, rhythm, wideness, and force (5-point Likert for each domain, from “Strongly disagree” to “Strongly agree”).
4. The CMPs’ and participants’ experiences were assessed using a questionnaire.

### Phase 2: Pilot

In the pilot phase, the APDC device was refined by the team using the same procedure as above. Details of the refinement can be found in our previous publication regarding the technical development of the ADPC [23], while this study focused on the users’ evaluation by participants and CMPs. A total of 42 participants’ pulse features were collected, and 10 CMPs each assessed 10‐11 participants’ pulse (a single participant’s pulse was assessed by 2 to 3 CMPs) and APDC-regenerated pulse and rated the agreement on the likelihood. They were asked to provide their feedback. The CMPs and participants were recruited from March 1-14, 2025.

### Participants Recruitment

The study included participants who met specific inclusion criteria: individuals aged between 18 and 65 years, with a BMI ranging from 18.5 to 30 kg/m², and those willing to provide informed consent. Participants were excluded if they had a dorsally located radial artery.

We recruited registered CMPs with at least 5 years of clinical experience from social media to assess the participants’ pulse and the machine-regenerated pulse. Afterward, they completed a predefined record form to evaluate the pulse characteristics.

### Tested Device

**Figure 1.**
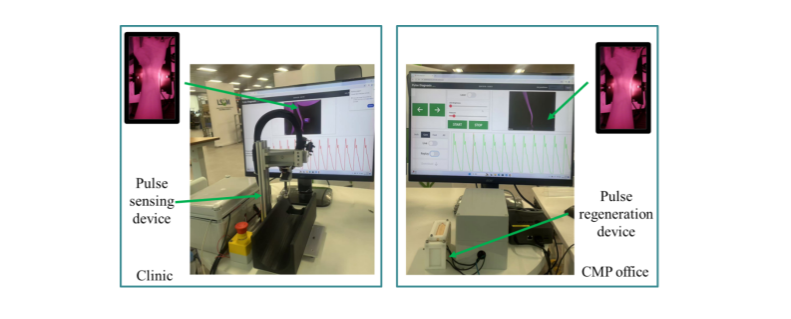
Overview of the proposed traditional Chinese medicine pulse–based tele-practice system. CMP: Chinese medicine practitioner.

A prototype of the APDC device (Logistics and Supply Chain MultiTech R&D Centre) was developed using a standard tele-practice infrastructure as an assistive technology for TCM pulse diagnosis (Figure 1). The system comprises 2 primary components: a pulse-sensing device at the clinic and a pulse-regeneration device at the TCM practitioner’s office. A pulse collection device was used at the patient’s location, featuring a screen to display pulse information. Correspondingly, at the CMP end, a pulse-regeneration device equipped with a screen displayed the transmitted data. Patients can place their arms on a pulse-sensing device. CMP can remotely control the device to adjust its position and precisely determine the optimal location for pulse sensing, as the accuracy of pulse diagnosis is highly dependent on proper positioning. Both modules feature digital interfaces for real-time data visualization, with the sensing unit displaying pulse data acquisition parameters and the regeneration unit showing the transmitted pulse characteristics. The sensing unit incorporates position-adjustable mechanisms that can be remotely controlled by TCM practitioners. This bidirectional setup enables patients to position their arms on the sensing platform, while allowing practitioners to optimize measurement locations remotely.

### Pulse Sensing Device

Palpation is a professional procedure used in TCM. Practitioners determine the palpation location on the participant’s arm and the applied force based on their expertise and experience [24]. The proposed pulse-sensing device features 2 key components: a radial artery viewing device and a dynamic force control, which assist practitioners in accurately determining the palpation location and the appropriate force to apply.

### Radial Artery Viewing Device

**Figure 2.**
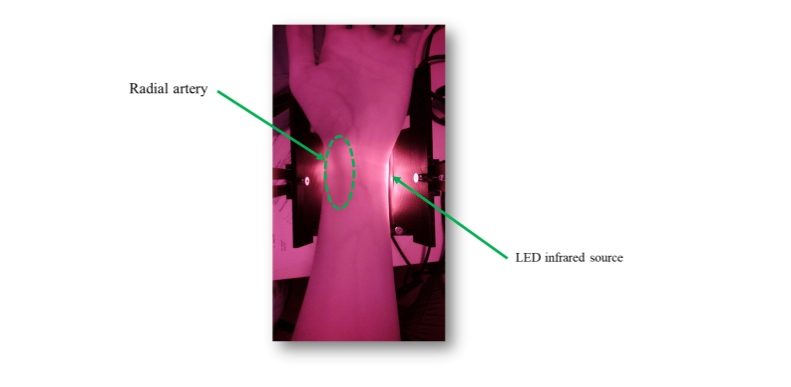
Infrared image of a participant’s arm showing the position of radial artery. LED: light-emitting diode.

**Figure 3.**
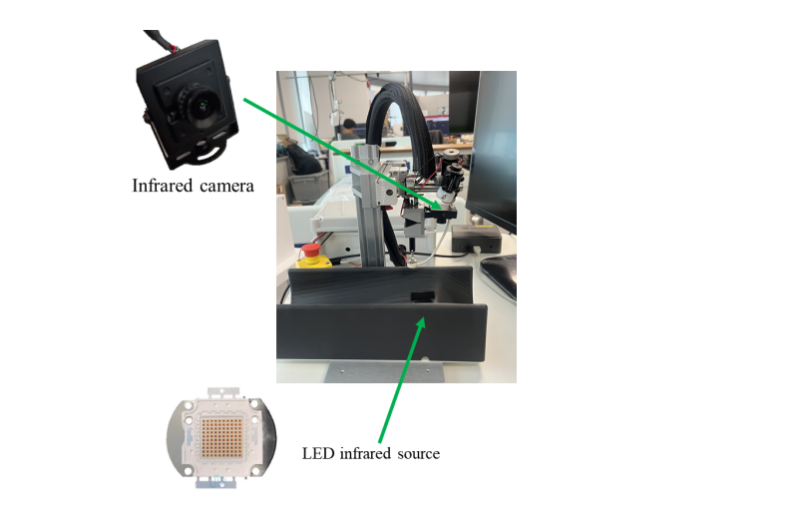
Key components of radial artery viewing device. LED: light-emitting diode.

The APDC system uses infrared technology to illuminate a patient’s arm and capture images using an infrared camera, as shown in Figure 2. Unlike the conventional blood vessel viewing device [25], the participants’ arm is positioned on a stabilizing device with an infrared light source beneath it and an infrared camera to capture the images shown in Figure 3.

### Pulse-Taking Procedure

The CMPs evaluated the participants’ pulse as they would in a clinical setting. The principal investigator assessed each participant’s pulse and used a marker pen to mark the radial pulse (cun, according to TCM theory). The technician then assessed the participant’s radial pulse, using the principal investigator’s mark as a guide, with the aid of infrared light, and began recording the pulse waveform data using the APDC device. The device recorded the participant’s pulse for 30 seconds at the wrist (*cun, guan,* and chi positions in terms of TCM) individually during the feasibility phase, and simultaneously during the pilot phase, after being upgraded to include 3 probes instead of a single probe based on feedback from CMPs during the feasibility phase.

After the APDC device recorded the pulse, the participant was seated on a chair with their arm resting on a table and their wrist positioned on a pulse pillow. The CMP assessed the pulse of the participant’s right or left hand for up to 1 minute each, as if in clinical practice, and recorded their pulse description on a chart. Subsequently, they assessed the regenerated pulse produced by the APDC device for up to 1 minute. Finally, they rated the agreement between the real participant’s pulse and the machine-generated pulse.

### Outcome Assessment

The user experience with the APDC device, which comprised a pulse collection device and a pulse re-generation device, was evaluated separately. Quantitative feedback from both participants and CMPs was gathered through a self-developed questionnaire. Qualitative feedback was collected through open-ended questions.

### Agreement Between Participants’ and Regenerated Pulse

CMPs were instructed to indicate their level of agreement with statements regarding the similarity between the machine-generated pulse and the actual pulse of participants. Responses were recorded using a 5-point Likert scale, ranging from 1 (Strongly Disagree) to 5 (Strongly Agree). The questionnaire evaluated 4 key pulse attributes: frequency, rhythm, wideness, and force. CMPs were asked to assess the extent to which they agreed that the frequency of the machine-regenerated pulse accurately resembled that of a real person’s pulse. Similarly, they evaluated the rhythm, wideness, and force of the machine-generated pulse in comparison to the actual pulse. This structured approach allowed for a comprehensive analysis of CMPs’ experiences and perceptions, contributing valuable data to the study of machine-regenerated pulse accuracy.

### CMPs’ Feedback on the Device

A self-developed questionnaire was used to evaluate CMPs’ comfort, ease of use, efficiency, and overall satisfaction with the machine. CMPs were asked to indicate their level of agreement with each statement using a 5-point scale. First, CMPs rated their comfort level during the measurement process, with options ranging from “Very uncomfortable” to “Very comfortable.” Second, they assessed the ease of understanding the instructions and using the machine, with responses from “Very difficult” to “Very easy.” Third, CMPs evaluated the efficiency of the measurement process, indicating their agreement with statements about its quickness and effectiveness, from “Strongly disagree” to “Strongly agree.” Finally, they expressed their overall satisfaction with the experience, choosing from “Very dissatisfied” to “Very satisfied.”

Additionally, qualitative feedback was collected through open-ended questions in a self-completed questionnaire, seeking insights on “How would you describe the overall experience of using the pulse regenerating device?” and “What were the most noticeable differences in terms of the pulse features between the machine-regenerated pulse and that of the participants?” and “If you could suggest improvements to the machine, what would they be?”

### Participants’ Feedback on the Device

Participants’ feedback on their experience with the pulse measurement process was collected. Participants were asked to indicate their level of agreement with various statements using a 5-point Likert scale. The questionnaire focused on 4 key areas: comfort during the measurement process, ease of understanding and using the machine, the efficiency of the measurement process, and overall satisfaction with the experience. It aimed to assess the user-friendliness and effectiveness of the machine from the Participants’ perspective.

Finally, qualitative feedback was gathered through open-ended questions in a self-completed questionnaire. Participants were asked to describe their overall experience with the pulse measuring device, suggest any improvements they would recommend for the machine, and share any additional insights or aspects of their experience that were not addressed in the previous questions. The questions used were: “How would you describe the overall experience of using the pulse-measuring device?” “If you could suggest improvements to the machine, what would they be?” and “Is there anything else you would like to share about your experience or any other aspect that was not covered in the previous questions?”

### Statistical Analyses

All analyses were performed using SPSS (version 28.0; IBM Corp). The CMPs’ and participants’ sociodemographic characteristics, including age and gender, presence of chronic diseases, and the CMPs’ rating on the likeliness between participants’ pulse features (frequency, rhythm, wideness, and force) and machine-regenerated pulse using descriptive statistics. Both parametric measures (mean [SD]) or nonparametric measures (median [IQR]) were reported accordingly due to the small sample size. Categorical variables (eg, gender and presence of chronic diseases) were expressed as frequencies and percentages (n, %).

### Ethical Considerations

The study was reviewed and approved by the Hong Kong Polytechnic University institutional review board (reference number HSEARS20240801005) and registered at ClinicalTrials.gov (Ref. NCT06974227). We recruited participants through social media and mass emails at the University. All participants signed a consent form to confirm their voluntary participation in pulse data collection. As an incentive, each participant received an HK $100 supermarket coupon (equivalent of US $12.81) upon completing either phase 1 or 2. Pulse recordings and associated demographic information were deidentified immediately after collection, and any potentially identifying information was removed or coded to safeguard participant privacy. All data were stored on encrypted, password-protected institutional servers accessible only to authorized research team members, and findings are presented in aggregate form to maintain confidentiality.

## Results

### Feasibility

During the feasibility phase, 5 CMPs and 10 participants were recruited. The 5 CMPs had a mean age of 36 (SD 6.2) years, 3 (60%) were female, and had a mean clinical experience of 11.2 (SD 6.3) years (Table 1). The 10 participants had a mean age of 41.1 (SD 15.2) years, 1 female (10%), and a mean BMI of 23.7 (SD 3.2) kg/m^2^ (Table 2).

**Table 1.** Chinese medicine practitioners’ characteristics and rating.

Variables	CMP^a^ (feasibility phase; n=5)	CMP (pilot phase; n=10)	
Age (years)			
Mean (SD)	36.0 (6.2)	41.5 (5.9)	
Median (IQR)	34 (9)	43 (7.5)	
Sex, n (%)			
Female	3 (60)	6 (60)	
Education level, n (%)			
Tertiary education	0 (0)	1 (10)	
Master’s degree	4 (80)	5 (50)	
Doctoral degree	1 (20)	4 (40)	
Clinical experience			
Mean (SD)	11.2 (6.3)	16.0 (5.8)	
Median (IQR)	10 (12)	19 (7.5)	
Agreement on Machine-regenerated Pulse (5=Totally agreement, 1=Totally disagreement)			
Frequency			
Mean (SD)	4.1 (0.58)	4.3 (0.72)	
Median (IQR)	4 (0)	4 (1)	
Rhythm			
Mean (SD)	3.8 (0.58)	4.0 (0.82)	
Median (IQR)	4 (0)	4 (0.25)	
Pulse width			
Mean (SD)	2.7 (0.88)	3.0 (0.94)	
Median (IQR)	3 (1)	3 (2)	
Pulse force			
Mean (SD)	2.4 (0.95)	2.8 (0.94)	
Median (IQR)	2 (1)	3 (1)	
Experience with Machine-generated Pulse Measurement (5=Totally agreement, 1=Totally disagreement)			
Comfort level			
Mean (SD)	3 (0.71)	3 (0.82)	
Median (IQR)	3 (1)	3 (2)	
Clarity of instructions on using the machine			
Mean (SD)	3.8 (1.30)	4.3 (0.95)	
Median (IQR)	4 (2.5)	4.5 (1)	
Efficiency			
Mean (SD)	2.2 (0.45)	2.5 (0.53)	
Median (IQR)	2 (0.5)	2.5 (1)	
Overall satisfaction			
Mean (SD)	2.6 (0.55)	2.6 (0.52)	
Median (IQR)	3 (1)	3 (1)	

a CMP: Chinese medicine practitioner.

**Table 2.** Participants’ characteristics and rating.

Variables	Participants (feasibility phase; n=10)	Participants (pilot phase; n=42)
Age (years)		
Mean (SD)	41.1 (15.2)	41.6 (13.3)
Median (IQR)	31.5 (31)	38.5 (22.75)
Sex, n (%)		
Female	1 (10)	28 (67)
BMI (kg/m^2^)		
Mean (SD)	23.66 (3.2)	21.6 (2.1)
Median (IQR)	23.96 (3.76)	21.48 (2.07)
Participants’ experience with the device (score range 1‐5), (5=Totally agreement, 1=Totally disagreement)		
Comfort level		
Mean (SD)	4.1 (0.74)	4.0 (0.66)
Median (IQR)	4 (0.25)	4 (0.25)
Ease of using		
Mean (SD)	4.1 (0.74)	3.9 (0.82)
Median (IQR)	4 (0.25)	4 (0.25)
Efficiency		
Mean (SD)	3.8 (0.92)	3.6 (0.85)
Median (IQR)	4 (1.25)	4 (1)
Overall satisfaction		
Mean (SD)	4.1 (0.74)	4 (0.68)
Median (IQR)	4 (1.25)	4 (1)

### CMPs’ Rating on Machine-Regenerated Pulse at Feasibility Phase

Five CMPs each evaluated pulses of 10 participants and the participants’ machine-generated pulses in terms of frequency, rhythm, width, and force. The results indicated an average agreement score of 4.1 (SD 0.6) for “frequency,” suggesting consensus among CMPs that the machine’s pulse frequency was consistent with the participants’ pulses. The “rhythm” dimension obtained an average score of 3.8 (SD 0.6), demonstrating that CMPs tended to rate this parameter with moderate agreement. “Pulse width” received a lower average score of 2.7 (SD 0.9), reflecting CMPs’ neutral to general agreement regarding this characteristic. The lowest average score of 2.4 (SD 1) was observed for “pulse force,” indicating a slight leaning toward disagreement among the CMPs.

### CMPs’ Experience With Machine-Generated Pulse Measurement at Feasibility Phase

CMPs reported their “comfort level” during the measurement process with an average score of 3 (SD 0.7), indicating a neutral perception. The “clarity of instructions on using the machine” received an average score of 3.8 (SD 1.3), demonstrating relatively straightforward operational procedures. Regarding “efficiency,” the significantly lower average score of 2.2 (SD 0.5) reflected moderate dissatisfaction among CMPs at the feasibility phase. Overall satisfaction with the measurement system yielded an average score of 2.6 (SD 0.6), suggesting a neutral appraisal of the machine-generated pulse assessments.

### CMPs’ Qualitative Feedback at the Feasibility Phase

#### Overall Perception

##### Performance in Pulse Reproduction

CMPs generally acknowledged that machines can reproduce pulse rate and rhythm with relative accuracy. However, simulations of pulse width and strength were suboptimal, prone to misjudgment, and occasionally exhibited asynchronous performance across the 3 pulse locations (cun, guan, and chi). This is particularly true in complex pulse characteristics during regeneration, inconsistencies in pulse strength variations, and notable deficiencies in detailed feature representation.

##### Usability Versus Diagnostic Precision

The CMPs deemed the device was user-friendly, although the devices demonstrated compromised overall accuracy, particularly in discerning subtle variations in pulse position intensity.

### CMPs’ Suggestion for Improvement After the Feasibility Phase

#### Temporal Continuity and Nuance Preservation

The machine-generated pulse showed discontinuities during pulse cycle transitions (eg, last-to-first beat replay artifacts), which required mitigation through extended sampling durations (>60 s) to minimize transitional irregularities.

#### Interactive Visualization Enhancement

Implementation of multimodal feedback systems is proposed, particularly visual documentation of contextual factors (ambient conditions and patient posture) during acquisition phases to compensate for limitations in real-time pulse regeneration fidelity.

### Participants’ Experience With the Device at the Feasibility Phase

Regarding comfort during the measurement process, participants reported their “comfort level” with an average score of 4.1 (SD 0.7), indicating they felt comfortable. The ease of using the machine was rated at 4.1 (SD 0.7), suggesting the participants found the process easy to follow. For the efficiency of the measurement process, the average score was 3.8 (SD 0.9), reflecting participants’ general agreement. Overall satisfaction received a score of 4.1 (SD 0.7), showing participants were satisfied with the machine-generated pulse measurement.

### Participants’ Qualitative Feedback at the Feasibility Phase

#### Overall Perception

##### Comfort

Most users found the device generally comfortable to use without significant discomfort, but it required strict wrist positioning and took some time to adjust.

##### Efficiency

The participants described the device as fast and efficient with easy-to-understand instructions. However, some participants encountered difficulties in locating accurate pulse positions, leading to prolonged measurement times.

##### Temperature Perception

The metal components felt colder compared to the warmth of human hands, although this did not cause major discomfort.

### Participants’ Suggestions for Improvement After the Feasibility Phase

#### Positioning Accuracy

Participants recommended integrating higher-precision sensors to enable automatic pulse localization, reducing the need for repeated positioning attempts.

#### Design

The participants also suggested the appearance can be improved. Examples included the exposed internal components and wires housing.

#### Comfort Optimization

The participants suggested incorporating padded cushions or cotton layers at wrist support areas and considering stabilizing straps to prevent displacement.

#### Adjustability Features

The participants suggested developing height-adjustable mechanisms for devices or seating to accommodate users’ optimal measurement postures.

### Pilot

After receiving feedback from the CMPs and participants at the feasibility phase, the team had refined the prototype of the device. The changes include “to increase the comfortability of arm rest from fixed to slidable and lockable when taking pulse,” “to enhance initial pressure adjustment to improve the overall experience by adjusting relative pressure to static and dynamic pressure,” “to refine dynamic pulse replication algorithms across cun-guan-chi to achieve clinical-grade realism in machine-generated pulse from “no achievable” to “in phase pulse waveforms’.” The details of the changes are presented in Table 3.

**Table 3.** Changes in the prototype according to Chinese medicine practitioners’ and participants’ feedback.

Parts	Feedback from CMPs^a^ or participants	Initial prototype (feasibility phase)	Final prototype (pilot phase)
Arm rest	Participants suggested the armrest needs to increase its comfortability when taking pulse	Fixed	Slidable and lockable
Pulse sensing technology	CMPs proposed optimizing multisensor arrays in pulse-taking devices to minimize signal artifacts during radial artery data acquisition.	Piezoelectric	Piezoresistive
Sensing parameter	Participants suggested enhancing initial pressure adjustment to improve the overall experience.	Relative pressure	Static and dynamic pressure
Number of pulse sensors	CMPs recommended using instruments equipped with simultaneous 3-finger pulse palpation to collect pulse data.	1	3
Force control technology	CMPs suggested enhancing sensor configurations in pulse-simulating devices to improve the accuracy of pulse waveform replication.	Load cell	Pulse sensor
Silicone skin	CMPs recommended integrating skin-like biomimetic materials into pulse-simulating devices to enhance the fidelity of pulse waveform reproduction	Off the shelf	Custom made
Three-pulse regeneration synchronization	CMPs emphasized refining dynamic pulse replication algorithms across cun-guan-chi to achieve clinical-grade realism in machine-generated pulse	Not achievable, as only one probe was used.	In-phase pulse waveforms are achieved by using 3 probes to collect pulses simultaneously at 3 positions.

a CMP: Chinese medicine practitioner.

### CMPs and Participants’ Characteristics

During the pilot phase, 10 CMPs and 42 participants were recruited. The 10 CMPs had a mean age of 41.5 (SD 5.9) years, 6 (60%) were female, and had a mean clinical experience of 16 (SD 5.8) years. The 42 participants had a mean age of 41.6 (SD 13.3) years, 28 (66.7%) were female, and had a mean BMI of 21.6 (SD 2.1) kg/m^2^.

### CMPs’ Agreement on Machine-Regenerated Pulse at Pilot Phase

Ten CMPs each evaluated 10 or 11 participants’ pulses and their machine-generated pulses in terms of frequency, rhythm, wideness, and force. The results indicated that the average agreement score for frequency was 4.3 (SD 0.7), suggesting that CMPs found the machine’s pulse frequency to be in agreement. Similarly, the rhythm received an average score of 4 (SD 0.8), indicating that CMPs perceived the rhythm as generally agreed. The wideness of the pulse was rated with an average score of 3 (SD 0.9), reflecting CMPs’ views that it had general agreement. Finally, the force of the pulse garnered an average score of 2.8 (SD 0.9), showing that CMPs felt the force reached general agreement.

### CMPs Experience With Machine-Generated Pulse Measurement at Pilot Phase

CMPs reported their “comfort level” during the measurement process with an average score of 3 (SD 0.8), indicating that they felt neutral. The “clarity of instructions on using the machine” was rated at 4.3 (SD 1), suggesting that CMPs found the process easily understandable. The “efficiency” of the measurement process was scored at 2.5 (SD 0.5), reflecting CMPs’ views that it was less than satisfactory. Overall satisfaction with the experience was rated at 2.6 (SD 0.5), showing that the CMPs held neutral views regarding the machine-generated pulse measurement.

### CMPs’ Qualitative Feedback at the Feasibility Phase

#### Overall Experience

##### User Experience and Comfort

CMPs reported the device’s rigid surface texture needed excessive palpation effort, with inaccuracy in the regeneration pulse at sunken (chen 沉), thready (xi細), and deficient (xu 虛) pulse regeneration.

##### Pulse Accuracy

While capable of replicating pulse frequency, rhythm, and width, the device underperforms in rendering vascular tension, directional force (maishi 脈勢), and pliability. Approximately more than half of overall pulse morphology can be simulated, although individual variations in cun-guan-chi

##### Gender Impact

The device demonstrated better pulse reproduction for male participants than female participants, probably due to probe dimensions and positioning protocols, warranting anthropometric optimization through future adjustment.

### CMPs’ Suggestion for Improvement After the Pilot Phase

#### Operational Principles and Usability Enhancements

Stratified depth samples of the pulses feature can be collected for CMPs’ comparison. The fixed cun-guan-chi spacing requires anthropometric re-engineering for clinical applicability. Noise arising from the motorized component should be reduced.

#### Automation and Ergonomic Optimization

They suggested developing automated cun-guan-chi localization algorithms and proposed frame expansion for enhanced ergonomics and upgraded depth sensing capabilities. To eliminate “pulsatile extrusion” artifacts, hemodynamic fluidity using algorithmic refinement can be explored. They also suggested implementing triple-finger span adjustability for anatomical adaptability.

#### Material Science Considerations

Optimize material compliance to mimic cutaneous pliability. Enhance vascular compliance in tubing systems to improve rendering of pulse turgor and tension states.

### Participants’ Experience With the Device at Pilot Phase

For comfort during the measurement process, participants reported their “comfort level” with an average score of 4 (SD 0.7), indicating that they felt comfortable. The “ease of using” the machine was rated at 3.9 (SD 0.8), suggesting that participants found the process easy to operate. Regarding the “efficiency” of the measurement process, the average score was 3.6 (SD 0.9), reflecting participants’ views that it met expectations. Overall satisfaction with the experience was rated at 4 (SD 0.7), showing that the participants were satisfied with the machine-generated pulse measurement.

### Participants Qualitative Feedback at Pilot

#### Overall Perception

##### Positioning Time Consumption

Prolonged durations during locating the pulse position were noted, requiring multiple attempts to achieve the accurate pulse position due to stringent placement requirements.

##### Pulse Taking Procedure

Strict postural immobilization requirements posed challenges. Compared to the device, face-to-face CMPs’ manual palpation, particularly regarding pressure modulation subtlety, is more comfortable.

##### User-Driven Optimization Proposals

Implementation of graduated pressure escalation protocols (low initial force with progressive intensification) is advocated to optimize procedural comfort.

### Participants’ Suggestions for Improvement After the Pilot Phase

#### Comfort and Efficiency

The device was acknowledged as comfortable in general and operationally efficient. However, the participants suggested the probe interface can be optimized to enhance comfort. They suggested integrating padded cushions at wrist support interfaces and using probes with materials that will not induce cold feeling when touching the skin.

#### Pulse Localization

Participants reported the technicians faced persistent challenges in achieving accurate localization. Sensor precision upgrades enabling automated localization algorithms were strongly recommended to reduce multiattempt positioning requirements.

#### Device Appearance

The participants deemed that the appearance of the device could be further improved, and the hand placement space could be expanded.

## Discussion

### Principal Findings

TCM pulse diagnosis with modern tele-practice technology presents unique challenges in preserving diagnostic authenticity. Our innovative APDC device demonstrates the feasibility of remote pulse assessment through mechanical reproduction. The combination of noninvasive infrared vessel visualization and calibrated force control enables practitioners to locate and assess the cun-guan-chi positions with precision comparable to traditional palpation methods. This technical solution addresses a critical barrier in TCM tele-practice. In this study, the CMPs’ and participants’ feedback revealed that the APDC device was feasible in collecting pulse data and regenerating the pulse without major discomfort. The CMP deemed it easy to understand the instructions and use the APDC device. However, their rating on efficiency and overall satisfaction with the device per se fell slightly behind. The participants were satisfied with the device in terms of the comfort level and efficiency.

The COVID-19 pandemic highlighted the need for remote TCM diagnostic capabilities [26,27]. Pulse features have been regarded as important information for CMPs to make a proper diagnosis and formulate a treatment plan [7,28]. Our feasibility and pilot study data from the general public demonstrated both the use and limitations of remote pulse diagnosis. While the CMPs tended to agree that the device resembled the pulse feature in frequency and rhythm, they also pointed out the limitation of the device in reproducing the feature in wideness and force. They also reflect their expectation for improvements in the accuracy of reproducing some complex pulse characteristics, such as the depth and directional force of the pulse. These findings indicate the need for enhanced capabilities in dynamic pulse variation capture.

From the participants’ perspective, the APDC device was generally deemed comfortable, easy to use, and efficient. Overall, the participants satisfied the APDC device performance. The participants’ suggestions in the pilot phase were that the probe should adjust the physical pressure while sensing the pulse. Our team has replaced the piezoelectric probe in the prototype with a piezoresistive probe, which was used in other similar devices [29,30] and can help reduce discomfort when exerting pressure on the participant’s wrist. The participants in both feasibility and pilot phases also suggested increasing the space and adding cushion pads to the hand placement rack. However, it may induce instability of the hand during pulse sensing. Therefore, we remade the rack into a slightly blended shape to fit the wrist posture. Future design can consider using straps to hold the hand position, but it may take longer time and reduce the efficiency of the device. In addition, our study found that the device performed better in male participants than in female participants. The lower agreement scores for pulse force and width observed among female participants may be influenced by the predominance of females in the pilot sample (9/15, 60%) as well as by sex-specific anatomical differences, such as a smaller radial artery diameter or differences in tissue compliance. Accordingly, future refinements incorporating anthropometric optimization are warranted to improve device performance across sexes. Moreover, in response to the qualitative feedback regarding the “cold feeling” and “rigid surface” of the probes, it would be appropriate to further discuss the necessity of adopting biomimetic or softer materials to better mimic human touch, as such material properties may directly influence practitioners’ tactile perception and user experience.

From the practitioners’ perspective, their overall satisfaction regarding the device did not improve and remained low in phase 2 even after adjustment. It is noteworthy that some key pulse features that are critically determinant in TCM pattern diagnosis were suboptimal, such as pulse width and strength [31]. Another issue was the pulse-taking process by the device that was deemed inefficient by the practitioners. Although we have pen-marked the participants’ cun position, the technician needs time to adjust the probes (pulse sensors) to collect the pulse at 3 positions. The practitioners suggested developing automated cun-guan-chi localization algorithms. In addition, while the 3 probes were fixed in positions, this may lead to some inaccuracy in the pulse data collection as the width between cun-guan-chi positions can be varied with the participants’ arm length. Therefore, some of the discrepancy between participants’ and machine-regenerated pulses can be explained by the variation due to probe placement rather than faulty mechanical regeneration, but we were unable to quantify how much disagreement was attributed to this issue. In the future, the probes should be improved by adjustable in-between width and explore the possibility of automatic locating the pulse position rather than manually.

TCM pulse diagnosis includes diverse variations that reflect complex pathological conditions, and multiple pulse characteristics can coexist [11,32,33]. Given the significantly lower scores observed for pulse force and pulse width, we acknowledge that the APDC device is currently limited to basic hemodynamic monitoring. Consequently, further adjustment and optimization are required to enhance the diagnostic fidelity and clinical applicability of the device. More clinical trials involving diverse patients with well-documented TCM diagnoses would be essential for validating the system’s efficacy across various TCM patterns and disease states. Blind tests can be done to have CMPs match the machine-regeneration pulse with the real participant’s pulse. Further systematic studies with larger, demographically diverse samples are needed to validate the accuracy of the device in the clinical implementation.

### Limitations

This study had some limitations. First, our participants were healthy volunteers recruited from nonclinical settings, without chronic illnesses or any acute or active disease conditions. As a result, the pulse characteristics examined may be less representative than those typically recorded by CMPs in clinical practice. Second, only 4 dimensions of the pulse were measured, and some complex pulse types, such as the tight pulse and soft pulse, require further assessment.

### Conclusions

This study demonstrates that an innovative wireless pulse sensing and regeneration system for TCM tele-practice is feasible. This technology enables CMPs to take remote pulse feature assessment. While promising, additional clinical validation is needed to address limitations in capturing complex and dynamic pulse patterns. Future research should focus on enhancing pattern recognition and regeneration capabilities to optimize the device’s application in TCM.

## Abbreviations

APDC: Assistive Pulse Data Collection
CMP: Chinese medicine practitioner
CONSORT: Consolidated Standards of Reporting Trials
TCM: traditional Chinese medicine
